# Unpacking the relation between children’s use of digital technologies and children’s well-being: A scoping review

**DOI:** 10.1177/13591045221127886

**Published:** 2022-09-22

**Authors:** Mattia Messena, Marina Everri

**Affiliations:** 8797 University College Dublin, Ireland

**Keywords:** digital technology negative use, technology use, Internet problematic use, technology addiction, risky online behaviours, children, well-being, scoping review

## Abstract

Over the last decade, a substantial number of studies have addressed children’s use of technologies and their impact on well-being. Nonetheless, there is still a lack of clarity on the operationalisation of technology use, well-being, and the relation between the two. This scoping review intended to shed lights on Digital Technologies Use, its operationalisation, and the relation between Digital Technologies Negative Use (DTNU) and children’s well-being. For the scope of the special issue we focused on negative use. Results showed two conceptualisations of DTNU: compulsive/addictive use of devices and the Internet (e.g., Internet addiction) and negative online experiences/risky behaviours (e.g., cyberbullying). Well-being in relation to DTNU was mainly studied in terms of psycho/social dimensions (e.g., depression), and a gap in cognitive well-being studies was identified. Study designs were largely quantitative, and, in most studies, well-being was considered as a predictor of DTNU. Also, research with children under 12 years was lacking. Future research on DTNU should look at: how dimensions of addiction and negative online experiences relate; provide more evidence on cognitive well-being; explore the interplay of well-being multiple components relying on integrative conceptual frameworks. The recent notion of digital well-being should also be explored considering the results of this review.

## Introduction

Over the last decade, the time children spend online has doubled and children’s Digital Technologies Use (DTU) has started at an earlier age (Eukidsonline, 2010-2020: Livingstone et al., 2011; Smahel et al., 2020). The recent Covid-19 pandemic has even increased children’s time online as an essential way not only to keep connections with their social contexts but also to give continuity to learning tasks (Nagata et al., 2020). Therefore, digital technologies have become an integral part of children’s daily activities: Socialization, education and entertainment are now also happening in the digital world (Navarro & Tudge, 2022).

A substantial number of studies have looked at children’s safety and protection related to DTU and their impact on children’s wellbeing, e.g. missing out on social experiences (Turkle, 2011), addiction to technological devices, and physical and mental health related problems (Twenge et al., 2018; Young, 1996). Despite the significant implications of these results for research and interventions, clarity on the definitions and operationalisations of Digital Technologies Negative Use (DTNU) seems to be lacking.

The notion of ‘use’ itself is problematic since it can include different aspects related to child-technology interaction: the types of devices used, the ways in which children use the devices and the Internet (frequency, time spent online), the type of activities (production, entertainment), the motives and the characteristics of the online experience, and the societal regulations (mediation processes, country legislation, etc.) (see Smahel et al., 2020).

Operationalisation problems also apply to the notion of well-being (Dickson et al., 2018; Dodge et al., 2012): Well-being is a multidimensional construct (Colombo, 1986) that encompasses positive mood, emotions, and self-evaluations (Diener et al., 1999), health, physical functioning, and absence of disease (Minkkinen, 2013), social acceptance and good quality of relationships with family and friends (Keyes, 1998). Research showed that DTNU can affect not only psychological and social dimensions, but also physical and cognitive dimensions of well-being (Iannitelli et al., 2018; Kwong & Fong, 2019). Studies on the relation well-being-DTU have pointed out that it is the intertwinement of individual, social and country level variables having an impact on well-being. These variables impact on children’s DTU, in terms of opportunities and risks that, in turn, affect short-term effects and long-term effects of well-being, and physical, psychological, and social aspects (Livingstone, 2016; Livingstone et al., 2015).

Taken together these studies signal a need for better understanding the conceptualisation and operationalisation of DTNU and well-being, as well as their relation while focussing on children’s perspectives (Dickson et al., 2018; Orben, 2020). In this sense, the purpose of this scoping review was to identify, illustrate and critically analyse the studies with school-age children that have addressed these aspects. This will allow scholars to rely on an overview of existing literature on the topic that can orient future research.

## Method

Scoping review method was chosen as a systematic and explorative method to search and analyse existing literature (see Munn et al., 2018).

### Study selection process

Three types of search terms were defined to identify studies focused on school-aged children, DTU and well-being (Table 1). Five databases were searched: Web of Science, Scopus, PsycInfo, ERIC International and IBSS, resulting in 2757 records. The search was carried out from January 2010 to June 2021 and limited to results in English or Italian language. After removing 687 duplicates, 2070 records were selected for the screening process.

**Table 1.** Systematic review search terms.

Children (title and abstract)	Child* OR youth OR teen* OR adolescen* OR minors OR kid* OR girl* OR boy* OR pupil* OR “school student*”
AND	
DTU (title and abstract)	"Internet use" OR "use of Internet" OR "smartphone use" OR "use of smartphone*" OR "ICTs use" OR "use of ICTs" OR "use of digital technolog*" OR "digital technolog* use"
AND	
Well-being (title and abstract)	Well-being OR "digital well-being" OR health OR “digital health”

### Screening and coding process

**Figure 1.**
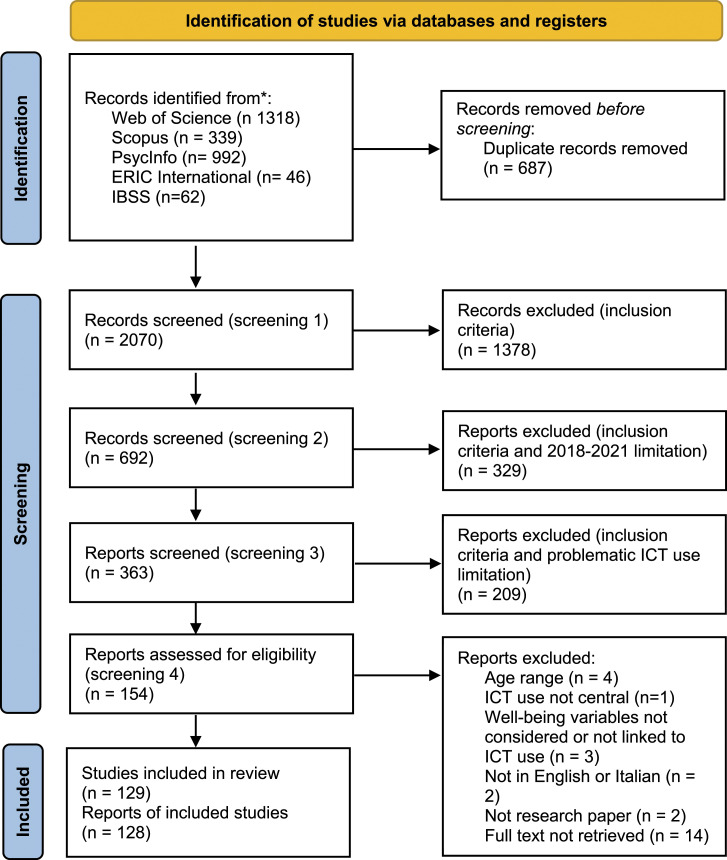
Search results presented using the PRISMA flow diagram (Page et al., 2021).

The screening was iterative rather than linear and was carried out in a four-step process (Figure 1).^1^ After the first screening of titles and abstracts which resulted in 692 articles, a further limitation was added, resulting in 363 records.^2^ Two reviewers screened titles and abstracts and independently coded the articles (Colquhoun et al., 2014; Levac et al., 2010). The operational definition of DTU was informed by the Activity Theory framework (Leont’ev, 1974) and its recent developments on technologies use (Everri, 2017; 2019; Lahlou, 2017; Yardi & Bruckman, 2011). In this framework, “technology [is considered] as part of the larger scope of human activity” (Kaptelinin & Nardi, 2006, p. 5). Therefore, for ‘use’ we intended every activity referred to or supported by digital technologies.

As for well-being, variables pertaining psychological (depression, anxiety), physical (sleep quality, dietary habits), social and cognitive (academic achievements) dimensions were included (Pollard & Lee, 2003). One-hundred-one studies were removed as they did not meet the inclusion criteria. Hence, two categories of studies on DTU were identified: (a) negative conceptualisations of use (*n* = 154), (b) ‘neutral’ conceptualisation of use (*n* = 108). For the topic of the special issue and for reason of space, this article will focus on the first category of studies (*N* = 154). The final sample consisted of 129 studies (see Figure 1).

### Data extraction

The conceptualisation of DTNU was extracted together with information related to the study construct operationalization when provided. Well-being dimensions were extracted and categorised following Pollard and Lee (2003).

## Results

Results are presented below and summary of the surveyed studies with the identified categories are reported in Table 2.^3^

**Table 2.** Summary of included studies (*N* = 129).

N	Authors	Year	Sample	Well-being	Digital technology negative use	Relation between variables
**1**	Al Majali, SA	2020	Children, pre-adolescents and adolescents	Psychological and social	Addictive use	Internet addiction was associated with emotional symptoms, peer relationship problems, and behavioural problems
**2**	Ali A., Horo A., Swain M.R., Gujar N.M., Deuri S.P.	2019	Adolescents	Psychological	Addictive use	Internet addiction was associated to depression, anxiety, and stress
**3**	Alt, D; Boniel-Nissim, M	2018	Adolescents	Psychological	Addictive use	Fear of missing out mediated the impact of parents’ positive communication on problematic internet use
**4**	Arrivillaga, C; Rey, L; Extremera, N	2020	Adolescents	Psychological	Addictive use	The impact of problematic Internet and smartphone use on suicidal ideation was moderated by emotional intelligence
**5**	Bai, C; Chen, XM; Han, KQ	2020	Pre-adolescents and adolescents from low-income families	Psychological, and cognitive	Addictive use	The impact of mobile phone addiction on school performance was mediated by depression
**6**	Bilgin, M; Sahin, I; Togay, A	2020	Adolescents	Social	Addictive use	Global distress level in the family impacted on social media disorder
**7**	Blinka, L; Sablaturova, N; Sevcikova, A; Husarova, D	2020	Adolescents	Psychological and social	Addictive use	Family and neighbourhood constraints impacted on the excessive Internet use after controlling emotional and behavioural problems
**8**	Boer, M; Stevens, GWJM; Finkenauer, C; de Looze, ME; van den Eijnden, RJJM	2021	Pre-adolescents and adolescents	Psychological and cognitive	Addictive use and negative experiences online	Social media use problems impacted on depression, life satisfaction, school achievements, and cybervictimization
**9**	Bolat, Nurullah; Yavuz, Mesut; Eliaçık, Kayı; Zorlu, Adil	2018	Adolescents	Psychological and social	Addictive use	Alexithymia positively and parents/peer attachment negatively predicted problematic Internet use
**10**	Brochado, S; Fraga, S; Soares, S; Ramos, E; Barros, H	2021	Adolescents	Psychological and social	Negative experiences online	Cybervictimization was associated with negative emotional well-being and school bullying victimization
**11**	Buctot, DB; Kim, N; Kim, JJ	2020	Adolescents	Psychological, physical, and social	Addictive use	Smartphone addiction significantly predicts health-related quality of life after accounting for demographic profile, family environment, and smartphone usage patterns
**12**	Cao, Ruilin; Gao, Tingting; Ren, Hui; Hu, Yueyang; Qin, Zeying; Liang, Leilei; Mei, Songli	2021	Pre-adolescents and adolescents	Psychological, physical, and social	Addictive use	The impact of bullying victimization on depression was mediated by Internet addiction and sleep quality
**13**	Casalo, LV; Escario, JJ	2019	Adolescents	Social	Addictive use	Parents’ care and knowledge impacted on excessive Internet use
**14**	Cerutti, R.; Spensieri, V.; Amendola, S.; Presaghi, F.; Fontana, A.; Faedda, N.; Guidetti, V	2019	Pre-adolescents and adolescents	Physical	Addictive use	The impact of problematic Internet use on somatic symptoms was mediated by sleep disturbance
**15**	Chang, Fong-Ching; Chiu, Chiung-Hui; Chen, Ping-Hung; Chiang, Jeng-Tung; Miao, Nae-Fang; Chuang, Hung-Yi; Liu, Shumei	2019	Children and pre-adolescents	Psychological, social, and cognitive	Addictive use	Poor academic performance, low household income, and depression were risk factors, whereas parent-child attachment was protective factor of smartphone addiction
**16**	Chao, CM; Kao, KY; Yu, TK	2020	Adolescents	Psychological and physical	Addictive use	Mental and physical health (in terms of cyberbullying, Internet pornography, Internet fraud) impacted on problematic Internet use
**17**	Charmaraman, L; Richer, AM; Moreno, MA (study 1)	2020	Adolescents	Psychological	Addictive use	High-risk gaming impacted on depressive symptoms and problematic internet behaviours
**18**	Charmaraman, L; Richer, AM; Moreno, MA (study 2)	2020	Adolescents	Physical	Negative experiences online	High-risk gaming impacted on sleep quality and skipping meals
**19**	Chau, Chor-lam; Tsui, Yvonne Yin-yau; Cheng, Cecilia	2019	Children and pre-adolescents	Psychological	Addictive useonline experiences	Reduction in Internet gaming disorder symptoms and risky online behaviours were associated to higher levels of positive affect and lower levels of negative affect
**20**	Chen, QQ; Lo Camilla, KM; Zhu, YH; Anne, C; Ling, CK; Patrick, I	2018	Adolescents	Psychological, physical, and social	Negative experiences online	Cybervictimization was positively correlated to symptoms of post-traumatic stress disorder, depression, self-harm, and other family-related (e.g. low income, child maltreatment) and physical variables
**21**	Cheung, JCS; Chan, KHW; Lui, YW; Tsui, MS; Chan, C	2018	Pre-adolescents and adolescents	Psychological	Addictive use	Self-esteem, loneliness and depression levels impacted on Internet addiction
**22**	Chiang, Jeng-Tung; Chang, Fong-Ching; Lee, Kun-Wei; Hsu, Szu-Yuan	2019	Children	Psychological	Addictive use	Depression predicted smartphone addiction
**23**	Choi, BY; Huh, S; Kim, DJ; Suh, SW; Lee, SK; Potenza, MN	2019	Adolescents	Psychological	Addictive use	Depressive, motor impulsive, smartphone addiction tendencies, and hyperkinetic attention-deficit/hyperactivity disorder (ADHD) predicted problematic Internet use
**24**	Chu, XW; Fan, CY; Lian, SL; Zhou, ZK	2019	Adolescents	Psychological	Negative experiences online	Self-esteem, social anxiety, and loneliness were predictors of cyberbullying victimization
**25**	Chwaszcz, J; Lelonek-Kuleta, B; Wiechetek, M; Niewiadomska, I; Palacz-Chrisidis, A	2018	Adolescents	Psychological	Addictive use	Coping strategies mediated the impact of personality-related factors in the development of Internet addiction
**26**	Davey A., Nasser K., Davey S	2020	Adolescents	Psychological	Addictive use	Internet addiction and phubbing impacted on smartphone addiction which in turn impacted on self-control
**27**	de Santisteban, Patricia; Gámez-Guadix, Manuel	2018	Adolescents	Psychological	Negative experiences online	Voluntary sexting, being a cyber-victim and depression predicted being involved in online sexual solicitation and interactions with adults
**28**	Demirtaş, Ozgur Onder; Alnak, Alper; Coşkun, Murat	2021	Adolescents with ADHD	Psychological	Addictive use	Social phobia predicted higher level of problematic Internet use
**29**	Do, KY; Lee, KS	2018	Adolescents	Physical	Addictive use	Problematic Internet use affected sleep and indirectly affected oral health
**30**	Domoff, Sarah E.; Borgen, Aubrey L.; Foley, Ryan P.; Maffett, Anissa	2019	Children, pre-adolescents and adolescents	Physical	Addictive use	Strongest evidence emerged regarding the impact of excessive use of electronic devices (EUED) and sleep outcomes. Mixed evidence emerged regarding EUED and physical activity and obesity
**31**	Domoff, SE; Sutherland, EQ; Yokum, S; Gearhardt, AN	2020	Adolescents	Psychological and physical	Addictive use	Emotion regulation difficulties mediated the association between addictive phone use and dysregulated eating, restrained eating, and food addiction
**32**	Donald, JN; Ciarrochi, J; Parker, PD; Sahdra, BK	2019	Adolescents	Psychological	Addictive use	Compulsive Internet use predicted reductions in trait hope and self-esteem
**33**	Dong, HX; Yang, FR; Lu, XZ; Hao, W	2020	Children, pre-adolescents and adolescents	Psychological	Addictive use	Internet addiction was associated to depression, anxiety, and stress
**34**	El Asam, A; Samara, M; Terry, P	2019	Pre-adolescents and adolescents	Psychological and social	Addictive use	Conduct problems, hyperactivity, impact on daily life activities, depression and poorer physical health predicted problematic Internet use
**35**	Emirtekin, E; Balta, S; Sural, I; Kircaburun, K; Griffiths, MD; Billieux, J	2019	Adolescents	Psychological	Addictive use	The impact of emotionally traumatic experiences on problematic smartphone use was mediated by body image dissatisfaction, social anxiety, and depression
**36**	Emre, N; Edirne, T; Ozsahin, A	2021	Adolescents	Psychological and physical	Addictive use	Being depressed and Internet addicted impacted on the consumption of tobacco products
**37**	Esmaeilzadeh Azad, Mahtab; Amini, Mahdi; Lotfi, Mozhgan	2018	Female adolescent	Psychological and social	Addictive use	Internalizing/externalizing symptoms and social isolation impacted on Internet addiction
**38**	Faltynkova, A; Blinka, L; Sevcikova, A; Husarova, D	2020	Adolescents	Social	Addictive use	Optimal parenting lowered the risk of excessive Internet use
**39**	Fan, BF; Wang, WX; Wang, T; Xie, B; Zhang, HM; Liao, YH; Lu, CY; Guo, L	2020	Adolescents	Psychological and physical	Addictive use	Problematic internet use impact on depression was mediated by misuse of opioids
**40**	Farhangpour, P; Maluleke, C; Mutshaeni, HN	2019	Adolescents	Psychological	Negative experiences online	Cyberbullying affected self-confidence, depression, frustration, self-consciousness, and suicidal thoughts
**41**	Feng, Yonghui; Ma, Yutong; Zhong, Qisong	2019	Pre-adolescents and adolescents	Psychological	Addictive use	The impact of stress on Internet addiction was mediated by social anxiety
**42**	Fırat, Sümeyra; Gül, Hesna; Sertçelik, Mehmet; Gül, Ahmet; Gürel, Yusuf; Kılıç, Birim Günay	2018	Adolescents referred to a psychiatric clinic	Psychological	Addictive use	Somatization, interpersonal sensitivity and hostility symptoms predicted problematic smartphone use
**43**	Fuchs, M; Riedl, D; Bock, A; Rumpold, G; Sevecke, K	2018	Inpatient adolescents	Psychological	Addictive use, and negative experiences online	Suicidality, difficulties in establishing stable and consolidated identity, and peer victimization were associated to problematic Internet use
**44**	Fumero, A; Marrero, RJ; Voltes, D; Penate, W	2018	Adolescents	Psychological and social	Addictive use	Psychopathology, personality features, and self-esteem had a greater impact on Internet addiction than social difficulties, social skills, and positive family functioning
**45**	Gamez-Guadix, M; de Santisteban, P	2018	Adolescents	Psychological	Negative experiences online	Less conscientiousness, more extraversion, and more depressive symptoms predicted sexting
**46**	Gao, QF; Jia, G; Fu, E; Olufadi, Y; Huang, YL	2020	Pre-adolescents and adolescents	Psychological and social	Addictive use	Smartphone use disorder was negatively associated with self-control and perceived parent-adolescent relationship, and positively associated with sensation seeking, loneliness, and anxiety
**47**	Gao, QF; Sun, RM; Fu, E; Jia, G; Xiang, YH	2020	Pre-adolescents and adolescents	Psychological, physical, and social	Addictive use	A good parent-child relationship and quality of life impacted on smartphone use disorder
**48**	Gao, TT; Li, MZ; Hu, YY; Qin, ZY; Cao, RL; Mei, SL; Meng, XF	2020	Adolescents	Psychological, physical, and social	Addictive use	Smoking, sleep deprivation and quality of parent-child relationship impacted on Internet addiction, depression and anxiety
**49**	Garaigordobil, M; Machimbarrena, JM	2019	Children, pre-adolescents and adolescents	Psychological	Negative experiences online	Both cybervictimization and cyberbullying perpetration were associated to high stress. Perpetrators had higher school stress; victims had higher scores in internalizing and externalizing emotional and behavioural problems
**50**	Giordano, C; Coco, GL; Salerno, L; Di Blasi, M	2021	Adolescents	Psychological	Addictive use	Emotional dysregulation affected problematic smartphone use
**51**	Gonzalez-Cabrera, J; Machimbarrena, JM; Fernandez-Gonzalez, L; Prieto-Fidalgo, A; Vergara-Moragues, E; Calvete, E	2021	Adolescents	Psychological, physical, social	Addictive use and negative experiences online	Lower quality of life related to psychological, physical and social factors was associated to accumulation of risk related to problematic Internet use, cybervictimization, sexting, and online grooming
**52**	Gonzalez-Cabrera, J; Touron, J; Machimbarrena, JM; Gutierrez-Ortega, M; Alvarez-Bardon, A; Garaigordobil, M	2019	Gifted children, pre-adolescents and adolescents	Psychological, physical, and social	Negative online experiences	The gifted sample presented more cybervictimization than cyberbullying, and cybervictimization was associated to worse health-related quality of life, depression, life satisfaction and stress
**53**	Gul, H; Firat, S; Sertcelik, M; Gul, A; Gurel, Y; Kilic, BG	2019	Clinical adolescents	Psychological	Addictive use and negative online experiences	Problematic smartphone use, lack of emotional awareness and e-bullying predicted e-victimization. Hostility, e-victimization and emotional awareness increased the risk of being an e-bully
**54**	Guo, L; Luo, M; Wang, WX; Huang, GL; Xu, Y; Gao, X; Lu, CY; Zhang, WH	2018	Adolescents	Psychological, physical	Addictive use	Problematic Internet use impacted on suicidal ideation and attempt through sleep disturbance. Sleep disturbance mediated the impact of suicidal behaviours on problematic Internet use
**55**	Heo, YoungJin; Lee, Kyunghee	2018	Adolescents	Psychological and social	Addictive use	Smartphone addiction impacted on school adjustment. Self-control partially mediated the relation in not at-risk subjects
**56**	Holfeld, Brett; Stoesz, Brenda; Montgomery, Janine	2019	Pre-adolescents and adolescents with autistic spectrum disorder	Psychological	Negative experiences online	Cyberbullying and cyber-victimization were associated with increased symptoms of anxiety but not depression
**57**	Hong, Ji Sun; Kim, Sun Mi; Kang, Kyoung Doo; Han, Doug Hyun; Kim, Jeong Soo; Hwang, Hyunchan; Min, Kyoung Joon; Choi, Tae Young; Lee, Young Sik	2020	Male adolescents with Internet gaming disorder	Psychological and physical	Addictive useonline experience	Physical exercise intervention in combination with cognitive behavioural therapy for individuals with Internet gaming disorder improved the severity of problematic internet use and depressive mood
**58**	Huang, Y; Xu, L; Mei, Y; Wei, Z; Wen, HY; Liu, DG	2020	Adolescents	Psychological	Addictive use	The impact of problematic Internet use on suicidal ideation was moderated by mood disorders, quality of life, impulsivity, and aggression
**59**	Khalil, SA; Kamal, H; Elkholy, H	2022	Adolescents	Psychological	Addictive use and negative online experiences	Depression, dysthymia, suicide, social anxiety panic, and phobias were associated to problematic Internet use, gaming disorder and Facebook addiction
**60**	Khasmohammadi, M; Ehsaei, SG; Vanderplasschen, W; Dortaj, F; Farahbakhsh, K; Afshar, HK; Jahanbakhshi, Z; Mohsenzadeh, F; Noah, SM; Sulaiman, T; Brady, C; Hormozi, AK	2020	Adolescents studying abroad	Psychological and social	Addictive use	Excessive Internet use and problem gambling impacted on psychological well-being through perceived peer support
**61**	Kheyri F., Azizifar A., Valizadeh R., Veisani Y., Aibod S., Cheraghi F., Mohamadian F	2019	Female adolescents	Psychological and cognitive	Addictive use	Positive and significant correlation between Internet dependency was positively associated to anxiety and negatively to academic performance
**62**	Kim, KM; Kim, H; Choi, JW; Kim, SY; Kim, JW	2020	Adolescents	Psychological	Addictive use	Depressive episodes, suicidal ideation, and suicidal attempts predicted problematic Internet use
**63**	Kim, Seung-Gon; Park, Jong; Kim, Hun-Tae; Pan, Zihang; Lee, Yena; McIntyre, Roger S	2019	Adolescents	Psychological	Addictive use	Attention-deficit hyperactivity disorder symptoms were risk factors for smartphone addiciton
**64**	Kircaburun, Kagan; Griffiths, Mark D.; Billieux, Joel	2020	Adolescents	Psychological	Addictive use	Childhood emotional maltreatment indirectly impacted problematic social media use (PSMU) via body image dissatisfaction (BID) among males. Only BID was positively impacted PSMU among females
**65**	Kojima, R; Sato, M; Akiyama, Y; Shinohara, R; Mizorogi, S; Suzuki, K; Yokomichi, H; Yamagata, Z	2019	Adolescents	Psychological, physical	Addictive use	Orthostatic dysregulation symptoms were predictor of problematic Internet use. Depression was predictor except among youngest students
**66**	Kokka, I; Mourikis, I; Nicolaides, NC; Darviri, C; Chrousos, GP; Kanaka-Gantenbein, C; Bacopoulou, F	2021	Pre-adolescents and adolescents	Physical	Addictive use	Problematic internet use was found to affect sleep quality and quantity and provoke insomnia symptoms
**67**	Lee J.-Y., Kim S.-Y., Bae K.-Y., Kim J.-M., Shin I.-S., Yoon J.-S., Kim S.-W	2018	Adolescents	Psychological and social	Addictive use	Academic stress, depression, and difficulties (e.g. peer problems) were predictors of problematic Internet use
**68**	Lee S.-Y., Lee D., Nam C.R., Kim D.Y., Park S., Kwon J.-G., Kweon Y.-S., Lee Y., Kim D.J., Choi J.-S	2018	Adolescents	Psychological and social	Addictive use	Problematic Internet and smartphone use were associated to addictive behaviours and other psychopathologies. Aggression and impulsivity were associated to problematic smartphone use in females
**69**	Lee, Eun Jee; Ogbolu, Yolanda	2018	Pre-adolescents	Psychological and physical	Addictive use	Depression but not sleep time impacted on smartphone addiction
**70**	Lee, Jeewon; Sung, Min-Je; Song, Sook-Hyung; Lee, Young-Moon; Lee, Je-Jung; Cho, Sun-Mi; Park, Mi-Kyung; Shin, Yun-Mi	2018	Adolescents	Psychological, social	Addictive use	Aggressive behaviours and self-esteem impacted on smartphone addiction
**71**	Lee, JY; Ban, D; Kim, SY; Kim, JM; Shin, IS; Yoon, JS; Kim, SW	2019	Adolescents	Psychological	Addictive use and negative experiences online	Problematic Internet use and cyberbullying impacted on psychotic-like experiences
**72**	Li, C; Liu, D; Dong, Y	2019	Adolescents	Psychological	Addictive use	Depression mediated the relation between self-esteem and problematic smartphone use. The influence of depression was moderated by interpersonal trust
**73**	Li, Dongfang; Guo, Yafei; Zhang, Lin; Tu, Mengjie; Yu, Quanlei; Li, Hongxia; Sun, Xiaojun; Jin, Shenghua	2020	Children, pre-adolescents and adolescents	Psychological and cognitive	Addictive use	Academic performance moderated the relation between preference for online social interactions and problematic Internet use
**74**	Li, JB; Mo, PKH; Lau, JTF; Su, XF; Zhang, X; Wu, AMS; Mai, JC; Chen, YX	2018	Adolescents	Psychological	Addictive use	A bidirectional causal association resulted between online social networking addiction and depression
**75**	Li, YC; Wang, Y; Ren, ZJ; Gao, M; Liu, QL; Qiu, CJ; Zhang, W	2020	Adolescents	Psychological, social	Addictive use	Cognitive function partially mediated the association between family and school pressures and Internet use disorder
**76**	Lim, Yangmi; Nam, Su-Jung	2020	Children, pre-adolescents and adolescents	Psychological	Addictive use	Problematic Internet use predicted depression
**77**	Lin, MP; Wu, JYW; You, JN; Hu, WH; Yen, CF	2018	Adolescents	Psychological and social	Addictive use	High impulsivity, depressive symptoms, low subjective well-being, and high virtual social support was all predictive of Internet addiction
**78**	Liu, CM; Liu, Z; Yuan, GZ	2020	Adolescents	Psychological	Addictive use and negative experiences online	Cyberbullying victimization was positively related to problematic Internet use through the mediating variables of mindfulness and depression
**79**	Mac Carthaigh, S; Griffin, C; Perry, J	2020	Adolescents	Physical	Addictive use	Sleep quality and quantity were weakly to moderately correlated to problematic smartphone use
**80**	Machimbarrena, JM; Gonzalez-Cabrera, J; Ortega-Baron, J; Beranuy-Fargues, M; Alvarez-Bardon, A; Tejero, B	2019	Pre-adolescents and adolescents	Psychological, physical and social	Addictive use	The severe problematic Internet use impacted on dimensions of health-related quality of life
**81**	Marengo, D; Settanni, M; Fabris, MA; Longobardi, C	2021	Pre-adolescents and adolescents	Psychological	Negative experiences online	Fear of missing out mediated the impact of peer exclusion from WhatsApp class group on emotional symptoms
**82**	Martinez-Ferrer, B; Moreno, D; Musitu, G	2018	Adolescents	Social	Addictive use	Problematic use of online social networking sites was associated to aggressive behaviours, and victimization
**83**	Martinez-Martinez, AM; Lopez-Liria, R; Aguilar-Parra, JM; Trigueros, R; Morales-Gazquez, MJ; Rocamora-Perez, P	2020	Adolescents	Psychological and cognitive	Negative experiences online	Cybervictimization impacted on academic performance through affecting emotional intelligence
**84**	Martin-Perpina, MD; Poch, FV; Cerrato, SM	2019	Children, pre-adolescents and adolescents	Psychological and social	Addictive use	Impulsiveness and perceiving a high level of family support together with a high use by parents and siblings were risk factors for excessive use of ICT
**85**	Mathew, P; Krishnan, R	2020	Adolescents	Psychological	Addictive use	Problematic Internet use was negatively associated to self-esteem
**86**	Mikuška, Jakub; Smahel, David; Dedkova, Lenka; Staksrud, Elisabeth; Mascheroni, Giovanna; Milosevic, Tijana	2020	Adolescents	Psychological and social	Addictive use	Positive family and school relationships negatively impacted on excessive Internet use through the mediation of emotional problems
**87**	Milani, Luca; La Torre, Giuseppe; Fiore, Maria; Grumi, Serena; Gentile, Douglas A.; Ferrante, Margherita; Miccoli, Silvia; Di Blasio, Paola	2018	Children, pre-adolescents and adolescents	Psychological and social	Addictive use and negative online experiences	Internet addiction and avoidant and distraction copying were risk factors for Internet gaming disorder, whereas quality of relations with teacher was a protective factor
**88**	Mo, Phoenix K. H.; Chan, Virginia W. Y.; Chan, Samuel W.; Lau, Joseph T. F	2018	Adolescents	Psychological and social	Addictive use	Social support was negatively related to emotion dysregulation and Internet usage, which in turn were positively related to Internet addiction
**89**	Mo, PKH; Li, JB; Jiang, H; Lau, JTF	2019	Adolescents	Psychological, physical, and social	Addictive use	Family support and depression mediated the association between problematic Internet use and smoking
**90**	Muchacki, M	2018	Adolescents	Psychological and social	Addictive use	Internet addiction was associated with lower quality of life
**91**	Muller, KW; Wolfling, K; Beutel, ME; Stark, B; Quiring, O; Aufenanger, S; Schemer, C; Weber, M; Reinecke, L	2018	Pre-adolescents and adolescents	Psychological	Addictive use	Low conscientiousness and high neuroticism predicted problematic Internet use, that was associated to stress and adjustment disorder symptoms
**92**	Nguyen, HTL; Nakamura, K; Seino, K; Vo, V	2020	Pre-adolescents and adolescents	Psychological, physical, and social	Negative experiences online	Cyberbullying predicted perceived academic pressure, unhealthy behaviours, school bullying, and risk of self-harm
**93**	Park, Min-Hyeon; Park, Subin; Jung, Kyu-In; Kim, Johanna Inhyang; Cho, Soo Churl; Kim, Bung-Nyun	2018	Adolescents	Psychological and physical	Addictive use	The effect of Internet use problems on sleep problems appeared to be different according to the presence or absence of the moderating effect of depression
**94**	Park, S; Lee, JH	2018	Adolescents	Physical	Addictive use	Problematic Internet use adversely affected oral health
**95**	Petruzelka B., Vacek J., Gavurova B., Kubak M., Gabrhelik R., Rogalewicz V., Bartak M	2020	Adolescents	Social	Addictive use and negative online experiences	Socio-economic status impacted on excessive internet use risk and excessive gaming risk
**96**	Przepiorka, A; Blachnio, A	2020	Adolescents	Psychological and physical	Addictive use	Depression positively predicted sleep problems through Facebook intrusion
**97**	Saikia, AM; Das, J; Barman, P; Bharali, MD	2019	Adolescents	Psychological	Addictive use	Internet addiction was associated with stress, depression, and anxiety
**98**	Sami, H; Danielle, L; Lihi, D; Elena, S	2018	Adolescents	Psychological and physical	Addictive use	The effect of sleep disturbances on suicidal ideation was moderated by internet addiction and mediated by depressive symptoms
**99**	Sarti, Daniela; Bettoni, Roberta; Offredi, Ilaria; Tironi, Marta; Lombardi, Elisabetta; Traficante, Daniela; Lorusso, Maria Luisa	2019	Adolescents with specific learning disabilities	Psychological and social	Addictive use	Problematic smartphone use was associated with reduced perception of social support and to a decreased ability to understand and solve social situations
**100**	Sela, Yaron; Zach, Merav; Amichay-Hamburger, Yair; Mishali, Moshe; Omer, Haim	2020	Adolescents	Psychological and social	Addictive use	The effects of low family expressiveness and high conflicts on problematic Internet use and time spent online are mediated by depression and fear of missing out
**101**	Seo, J; Lee, CS; Lee, YJ; Lee, MS; Bhang, SY; Lee, D	2020	Pre-adolescents and adolescents	Psychological and social	Addictive use	Depressive symptoms mediated the impact of adverse childhood experiences on problematic internet use
**102**	Setiadi, R; Tini, T; Sukamto, E; Kalsum, U	2019	Pre-adolescents	Psychological	Addictive use	Smartphone addiction impacted on emotional mental disorder
**103**	Settanni, M; Marengo, D; Fabris, MA; Longobardi, C	2018	Adolescents	Psychological	Addictive use	Attention-deficit and hyperactivity disorder (ADHD) symptoms positively predicted addictive Facebook use, past negative and present fatalistic orientation, and negatively predicted future orientation. Further, past negative and present fatalistic orientations mediated the relationship between ADHD symptoms and addictive Facebook use
**104**	Su, Binyuan; Yu, Chengfu; Zhang, Wei; Su, Qin; Zhu, Jianjun; Jiang, Yanping	2018	Pre-adolescents and adolescents	Social	Addictive useonline experiences	Father–child relationship had a reciprocal, indirect effect on the relationship between parental monitoring and Internet gaming disorder, while mother–child relationship did not
**105**	Sun, RM; Gao, QF; Xiang, YH; Chen, T; Liu, T; Chen, QY	2020	Pre-adolescents and adolescents	Social	Addictive use	The impact of parent-child relationships on mobile phone addiction tendency was partially mediated by psychological needs satisfaction and moderated by peer relationships
**106**	Szasz-Janocha, C; Vonderlin, E; Lindenberg, K	2021	Pre-adolescents and adolescents with Internet use disorder	Psychological	Addictive use	Reduction in Internet use disorder symptoms resulted associated to reduction in depression, social anxiety, performance anxiety and school anxiety
**107**	Takahashi, M; Adachi, M; Nishimura, T; Hirota, T; Yasuda, S; Kuribayashi, M; Nakamura, K	2018	Children and pre-adolescents	Psychological	Addictive use	Pathological and maladaptive Internet use increased severe depression and decreased health-related quality of life
**108**	Tenzin, K; Dorji, T; Choeda, T; Wangdi, P; Oo, MM; Tripathy, JP; Tenzin, T; Tobgay, T	2019	Adolescents	Psychological, physical, social, and cognitive	Addictive use	Internet addiction was associated to depression and anxiety, and affected academic performance, social interactions and sleep
**109**	Urbanova, LB; Holubcikova, J; Geckova, AM; Reijneveld, SA; van Dijk, JP	2019	Adolescents	Psychological and social	Addictive use	Life satisfaction partially mediated the association between socio-economic status and excessive Internet use in the case of low perceived family wealth
**110**	van den Eijnden R.J.J.M., Geurts S.M., Ter Bogt T.F.M., van der Rijst V.G., Koning I.M.	2021	Pre-adolescents and adolescents	Physical	Addictive use	Problematic social media use predicted later bedtime, yet the effect of problematic social media use on the perceived quality of sleep was non-significant
**111**	Veisani, Y; Jalilian, Z; Mohamadian, F	2020	Adolescents	Psychological and social	Addictive use	The excessive use of internet, lack of control, and neglect social life were significantly correlated with mental health
**112**	Venuleo C., Ferrante L., Rollo S	2021	Adolescents	Psychological	Addictive use	Social anxiety, negative emotions, and loneliness were risk factors for problematic Internet use
**113**	Wang, C; Li, KG; Kim, M; Lee, S; Seo, DC	2019	Children, pre-adolescents and adolescents	Psychological	Addictive use	Excessive use of electronic devices was risk factor for psychological distress
**114**	Wang, Jiayi; Wang, Pengcheng; Yang, Xiaofan; Zhang, Guohua; Wang, XingChao; Zhao, Fengqing; Zhao, Meng; Lei, Li	2019	Adolescents	Psychological	Addictive use	Fear of missing out and procrastination sequentially mediated the relation between sensation seeking and smartphone addiction
**115**	Wang, ST; Xie, FZ	2021	Left-behind children, pre-adolescents and adolescents	Psychological and social	Addictive use	Pathological Internet use partially mediated the relation between perceived personal discrimination and problem behaviour. Emotional intelligence moderated both the relationship between perceived personal discrimination and problem behaviour, and the relation between pathological Internet use and problem behaviour
**116**	Wang, ST; Zhang, DM	2020	Pre-adolescents and adolescents	Psychological and social	Addictive use	Perceived personal discrimination mediated the relation between perceived social support and pathological Internet use. Emotional intelligence moderated both the relationship between perceived social support and perceived personal discrimination, and between perceived personal discrimination and pathological Internet use
**117**	Wartberg, L; Kammerl, R	2020	Adolescents	Psychological and social	Addictive use and negative online experiences	Antisocial behaviours, emotional distress, self-esteem problems and hyperactivity/inattention affected problematic use of technology. Anger control problems affected problematic gaming only
**118**	Wartberg, L; Zieglmeier, M; Kammerl, R	2021	Adolescents	Psychological and social	Addictive use and negative online experiences	Emotional distress predicted problematic Internet use; hyperactivity/inattention and self-esteem problems predicted problematic gaming; problematic gaming and hyperactivity/inattention predicted the co-occurrence of problematic gaming and problematic Internet use
**119**	Xiang, MQ; Lin, L; Wang, ZR; Li, J; Xu, ZB; Hu, M	2020	Pre-adolescents and adolescents	Psychological and physical	Addictive use	The relationship between sedentary behaviour and problematic smartphone use was moderated by self-control
**120**	Xie, XC; Dong, Y; Wang, JL	2018	Pre-adolescents and adolescents	Physical	Addictive use	Sleep quality mediated the relationship between problematic smartphone use and physical health symptoms
**121**	Xu, Ting-Ting; Wang, Hai-Zhen; Fonseca, Winny; Zimmerman, Marc A.; Rost, Detlef H.; Gaskin, James; Wang, Jin-Liang	2019	Adolescents	Psychological	Addictive use	Depressive symptoms mediated the association between academic stress and problematic smartphone usage. The degree of problem-focused coping moderated this relation
**122**	Yamada, M; Sekine, M; Tatsuse, T	2018	Children, pre-adolescents and adolescents	Physical and social	Addictive use	Late bedtime, physical inactivity and no family affluence impacted on prolonged screen time
**123**	Ying, CY; Awaluddin, SM; Kuay, LK; Man, CS; Baharudin, A; Yn, LM; Sahril, N; Omar, MA; Ahmad, NA; Ibrahim, N	2021	Adolescents	Physical	Addictive use	Internet addiction was associated with unhealthy dietary habits, sedentary behaviours, smoking and alcohol consumption
**124**	Youn, H; Lee, SI; Lee, SH; Kim, JY; Kim, JH; Park, EJ; Park, JS; Bhang, SY; Lee, MS; Lee, YJ; Choi, SC; Choi, TY; Lee, AR; Kim, DJ	2018	Adolescents	Social	Addictive use	Smartphone addiction correlated with isolated peer relationships
**125**	Yu, Yuelin; Yang, Xue; Wang, Suping; Wang, Huwen; Chang, Ruijie; Tsamlag, Lhakpa; Zhang, Shuxian; Xu, Chen; Yu, Xiaoyue; Cai, Yong; Lau, Joseph T. F	2020	Adolescents	Psychological and physical	Addictive useonline experiences	Insomnia and depression mediated the relation between Internet gaming disorder (IGD) and suicidal ideation. Insomnia partially mediated the association between IGD and depression
**126**	Zhai, BY; Li, DP; Li, X; Liu, YX; Zhang, JY; Sun, WQ; Wang, YH	2020	Children, pre-adolescents and adolescents	Psychological and social	Addictive use	Both school belonging and depressive symptoms mediated the link between perceived school climate and problematic Internet use
**127**	Zhai, ZW; Hoff, RA; Howell, JC; Wampler, J; Krishnan-Sarin, S; Potenza, MN	2020	Adolescents	Psychological and social	Addictive useonline experiences	At-risk problem gaming impacted on violent experiences (e.g. weapon-carrying, having been threatened with weapons, feeling unsafe at school, and serious fighting leading to injury)
**128**	Zhen, R; Liu, RD; Hong, W; Zhou, X	2019	Children, pre-adolescents and adolescents	Psychological and social	Addictive use	Parent-child relationship had a direct and negative effect on problematic mobile phone use (PMPU), and teacher-student relationship had an indirect effect on PMPU, both through the mediation of loneliness
**129**	Zorbaz, O; Demirtas-Zorbaz, S; Kilic, OU	2020	Adolescents	Psychological and social	Addictive use	Being ignored, exclusion, and daily hassles predicted problematic Internet use

### Sample characteristics

Most of the studies (86 out of 129) recruited samples of adolescent children in the age range 12–19 years. Excluding literature reviews, nearly all studies (*N* = 123) recruited a sample of both male and female, whereas two studies included only females, and one study included only males. Moreover, the large majority addressed a general sample of students (*N* = 111).

### Study design and methods

Quantitative research design represented most of the surveyed studies (*N* = 124): 109 were cross-sectional, 12 longitudinal, one pre-post-test experiment, one prospective trial, and one meta-analysis. Two were mixed-method studies, and three literature reviews. Overall, 104 studies presented causal relations, while 25 presented correlations.

### Definition and operationalization of children’s DTNU

Two strands of studies emerged from our analysis: (1) Studies that operationalised DTNU in terms of excessive use and addiction; (2) Studies that operationalised DTNU in terms of children’s risky /negative online experiences or behaviours.

#### DTNU as excessive use and addiction

A small number of studies (*N* = 4) used broad definitions of technologies (electronic devices, ICT, or screen devices) and measured DTNU with frequency of use indicators, where excessive use corresponded to ≥ 3 hours of use. Instead, a larger number of studies (*N* = 116) looked at the specific negative use of the Internet, of the smartphone, and of social network sites use (SNS). Studies on *negative use of the Internet* referred to the construct of Internet addiction. The most considered dimension of addiction (*N* = 58) was the interference of Internet use with daily life routines and tasks and the related disturbance of adaptive functions; followed by compulsion/loss of control (*N* = 49), and concern, fears or worries related to not being able to access the Internet (*N* = 43). A smaller number of studies considered, respectively, withdrawal and intolerance (*N* = 17), and social comfort/preference for online social interactions (*N* = 15). Nine studies included either escaping, namely using the Internet to change negative mood (*N* = 6), or feeling lonely, depressed, or moody (*N* = 3). In seven studies, the negative use of the Internet was also defined by problems with time management and relapse (i.e. trying unsuccessfully to spend less time on the Internet), whereas six studies included dimensions of addictive automatic thoughts/positive anticipation, and deviant behaviours. Other dimensions considered for Internet negative use were salience (skipping lunch or sleeping less because of the use of Internet) (*N* = 5), deficient self-regulation and conflicts (*N* = 4), lying to hide addictive behaviours and interpersonal and health-related problems (*N* = 3), primacy (i.e. Internet use occupies the centre of users’ thinking and behaviours), the physiological responses and negative emotions caused by relapse (i.e. unsuccessfully try to spend less time online), and anxiety/nervousness (*N* = 2). Only one study considered excessive gaming as part of Internet negative use. Continuation, obsession, excessive use, and addictive behaviours were dimensions studied separately in single studies.

Studies on the *negative use of the smartphone* operationalised DTNU using addiction dimensions. The most studied dimension was withdrawal (*N* = 23), followed by difficulties in daily living/disturbance for adapting functions (*N* = 18), tolerance (*N* = 16), and virtual orientation (*N* = 14). Six studies included the excessive use/overuse dimension, whereas the loss of control was considered in five studies. Symptoms of anxiety, escaping, loss of productivity, and compulsive behaviours were used in four studies, whereas physical symptoms and positive anticipation in three. Single studies included smartphone craving and peer dependence. One study used the concept of phubbing, i.e. disturbance in communication while being with others and obsession with the phone. Among the 11 studies that investigated the *negative use of SNS*, withdrawal symptoms were the most considered indicators of negative use (*N* = 10), followed by conflict with other people or activities (*N* = 9). Escaping and tolerance were included in six studies, whereas cognitive and behavioural salience and relapse in five. Persistence (i.e. being unable to stop using social media, even though others told the person that she really should), displacement (i.e. loss of interest in other activities), deception (using social media secretly), and preoccupation were included in four studies. Reinstatement, euphoria, loss of control were indicators of SNS negative use in three studies, whereas only one included compulsion.

#### DTNU as negative online experiences or risky behaviours

Another strand of studies on DTNU included studies on: (a) cyberbullying, (b) sexual solicitation online, sexting and cyberdating abuse, and (c) gaming. Most studies that referred to *DTNU as cyberbullying* focused on social exclusion dimensions (*N* = 8). One study addressed specifically the exclusion from WhatsApp classmate groups. Spreading rumours, lies or secrets was the second most considered dimension (*N* = 6), followed by visual forms of cyberbullying such as disseminating compromising or embarrassing photos or videos online (*N* = 5). Online threats were indicators of cyberbullying in 4 studies, whereas written forms of cyberbullying (e.g. aggressive/nasty texts), verbal forms (e.g. frightening phone calls), insults, and name-calling were reported as indicators in three studies. Slandering and uploading fake photos/videos online were included in two studies. Also, two studies considered sexual harassment as a dimension of cyberbullying. One study referred to impersonation.

The frequency of *sexual solicitations* and interactions online with an adult were experiences investigated in two studies, whereas sexting was addressed by three studies measuring the frequency of voluntarily sending text, photos, video, or sharing images via webcam with sexual content. One study defined the concept of *cyberdating abuse*, operationalizing it in terms of controlling the partner’s mobile phone and insulting.

Studies on *gaming* were 11 and ten operationalized the construct of negative use considering diagnostic criteria provided by the DSM V. One study referred to the Minnesota Impulsive disorder Interview which assesses a series of dimensions such as craving and neglecting other activities and being aware of having a problem with gaming.

### Children’s well-being and DTNU

*Psychological well-being* was the most studied aspect in relation to children’s DTNU (110 studies included at least one psychological variable). Social well-being variables were addressed in 54 studies, physical well-being in 35, and cognitive well-being in seven. Among the psychological well-being variables, depression or other mood variables (dysthymia) were the most investigated (41 out of 110 studies), as well as emotions and affection factors (emotion dysregulation, anger) (*N* = 22). Anxiety was addressed in 14 studies, and behavioural problems (e.g. internalizing/externalizing symptoms) in 14 studies. Stress-related factors were found in 13 studies, whilst self-harm or suicidal behaviours/ideation were included in 11. Fewer studies focused on life satisfaction or psychological components of quality of life (*N* = 10), self-esteem (*N* =9 ), fear of missing out (*N* = 4) and coping strategies (*N* = 2).

Fifty-four studies focused on *social well-being*: 16 studies considered family-related well-being variables (e.g., quality of parent-child relationship). Fourteen studies included peer-related variables (bullying/aggression or support); while 10 considered a broad definition of quality of life or well-being related to social factors. Five studies considered general social support, social isolation/dysfunction, and school-related well-being variables (e.g., teacher-student relationship, school adjustment). Antisocial behaviours were included in one study, and three studies dealt with stress or constraint factors related to the social context. Lastly, one study considered adverse childhood experiences.

Half of the studies on *physical well-being* focused on sleep-related factors such as sleep quality/duration or insomnia (18 out of 35). Nine studies included a physical component of well-being, six studies investigated well-being variables related to dietary habits and body shape (e.g., obesity), whereas five studies included factors related to substance abuse. Lastly, two studies dealt with somatic symptoms, other two studies investigated oral health factors. Cognitive well-being was the least investigated dimension: Six studies focused on school performance and one study was on academic achievements in relation to children’s DTNU. Interestingly, one study conceptualized mental health in terms of cyberbullying, Internet pornography and Internet fraud.

### The relation between children’s DTNU and well-being

Among the quantitative studies, 57 records considered well-being variables as predictors of children’s DTNU, whereas 38 considered well-being variables as an outcome. In the first group (*well-being as predictor*), 15 studies tested the mediation of other well-being variables, three of which proposed complex models with the moderation of a third variable. Lastly, one study addressed the moderation of self-control on the association between sedentary behaviours and smartphone addiction.

As for the second group (*well-being as outcome*), 11 studies tested the mediation of well-being variables, and two mediations were moderated by a third well-being variable; three studies investigated a moderated model. Moreover, three studies investigated well-being variables as both antecedents and outcomes with the mediation of DTNU variables: One tested the mediation of Facebook intrusion in the association depression-sleep problems, the other investigated the mediation of pathological Internet use in the association perceived personal discrimination-problem behaviours, and the third considered the mediation of Internet addiction in the relationship between bullying, victimization and psycho-physical well-being. One study investigated cyberbullying as the antecedent of negative Internet use with the mediation of depression.

Most correlational studies presented associations between well-being and excessive/addictive use (16 out of 25). Among them, physical and cognitive well-being dimensions were the least considered. Six studies presented correlations between psychosocial and physical well-being and negative experiences: For instance, cyberbullying experiences, sexting, and online grooming were associated with personality disorders, emotional symptoms, and behavioural problems. Additionally, three correlational studies focused on both excessive/addictive use and negative experiences. Three reviews looked at the impact of excessive/addictive use on physical wellbeing, in particular sleep quality and duration, physical activity and obesity, musculoskeletal outcomes/pain, ocular health, and migraine/headaches.

## Discussion and conclusion

Our literature review addressed the negative side of DTU in relation to children’s well-being to clarify the use of terms and leverage insight on the relation between DTNU and wellbeing. The conceptualisation of DTNU was twofold: one concerned excessive/addictive use of devices of the Internet and of social media platforms; the other referred to the children’s negative experiences or risky behaviours. The first conceptualisation was found in most of the studies surveyed and concerned a strand of studies on Internet and smartphone addiction, overuse, and the different nuances of dependency and compulsive behaviours. A minority of studies measured DTNU in terms of frequency of use/screen time. This indicates an important advancement in the field of DTNU research, since the frequency of use as an indicator of negative use has been extensively criticised (Orben, 2020; Sewall et al., 2020). In fact, most studies used operationalisations of the construct of technology addiction to refer to DTNU. Nonetheless, the concept of technology addiction continues to be problematic as it considers the Internet, the device, or the applications, as singular entities, although they afford an array of potential activities (Ryding & Kaye, 2018). In other words, the operationalisation of DTNU should not only address the type of medium used, but also the different possible activities supported by the medium, which could contribute to addictive behaviours. Researchers should further develop this line of investigation since the debate on Internet and smartphone addiction is vivid, not only in scientific communities, but also in the public discourse.

The second conceptualisation of DTNU concerned negative experiences of children with digital technologies, namely risky and harming behaviours such as cyberbullying, sexting, and gaming. Within an activity theory framework, the experiential aspect of use and the dependency deriving from the use can be linked as they are different components of an activity. Two recent studies have addressed these aspects by studying the relation between Internet addiction and children’s cybervictimization, sexting, and online grooming (González-Cabrera et al., 2021) and cyberbullying as the antecedent of Internet addiction (Liu et al., 2020). Moreover, as suggested by the recent developments of the activity theory (e.g., Lahlou, 2017) additional aspects should be considered such as the specific type of device/platform used and the societal regulations (e.g. parental mediation, legislation) in place.

As for the conceptualisation of well-being in relation to DTNU, the literature has largely addressed psychological individual dimensions with most studies focussing on depression and anxiety. Also, if some social and contextual dimensions have been considered (e.g., Mo et al., 2019; Mikuška et al., 2020), there is a neglect of studies that have included learning difficulties and school attainment variables. The relation between DTU and well-being is anything but simple and unidirectional: a higher number of studies considered well-being dimensions as predictors of DTNU; nonetheless, consistent studies have shown that DTNU can also contribute to the development of psychological problems. The most recent studies proposed mediation and moderation models in which different dimensions of well-being, various DTNUs as well as psychosocial variables (e.g., perceived discrimination) were included as mediators or moderators. This signals an emerging trend in the literature: the design of complex models which can account for the interplay of various dimensions when children’s use of technologies is concerned. Hence, ecological, and integrative conceptual frameworks (e.g., Hertlein, 2012; Livingstone et al., 2015) could become important points of reference for scholars interested in searching the area of DTU and well-being.

Elaborating further on DTNU and wellbeing-related outcomes, researchers should consider the recently developed notion of *digital well-being* (Gui et al., 2017; Vanden Abeele, 2021). Theoretically, digital well-being can derive from a plausible causal chain going from digital practices to well-being through experiences of harms and benefits (Büchi, 2020). This is an emerging line of inquiry which requires to be explored further in the light of the analysis of the studies presented in this review.

Lastly, one concluding remark on sampling and method. The vast majority of the surveyed studies recruited samples of adolescents, while studies with children under the age of 12 years were underrepresented; yet children’s DTU starts at an earlier age (Smahel et al., 2020). Future research should start involving younger children in research on DTU as well as devise methods suitable for this cohort of participants. In fact, self-reported measures of use were privileged in DTNU studies. This can be problematic not only for the accuracy of children’s estimate of the use (Sewall et al., 2020), but also for devising interventions based on children’s reports. Researchers should consider adopting research practices that resonate with children’s cultures of communication (Cristensen & James, 2017), as well as exploring emerging methods for data collection based on technologies’ features, e.g. applications on participants’ smartphones, for tracking use. Digital ethnography and participatory methods (Christen & James, 2017; Pink, 2016) can also be considered for actively involving children in the data collection, as well as for the combination with self-reported measures in mixed methods research designs (Everri, 2017). We hope that this review will contribute to clarifying a complicated but much needed line of inquiry as well as encouraging the design of further empirical works based on a child-centred approach.

## Notes

1 Inclusion criteria were as follows: (1) The sample included school age children (5–19 years); (2) The study design included DTU; (3) The study considered children’s wellbeing variables; (4) The study was a research contribution in the form of a journal article or proceeding paper.

2 Specifically, we noted that from 2018 to 2021, the number of references concerned with the searched topic doubled. For this reason, we decided to focus on the analysis of papers published within that time frame.

3 For word count limit we made the choice to not include the references of the screened articles in the presentation of the results. Table 2 presents a detailed categorization of the resources identified.
